# The miRNA mir-582-3p suppresses ovarian cancer progression by targeting AKT/MTOR signaling *via* lncRNA TUG1

**DOI:** 10.1080/21655979.2021.2003662

**Published:** 2021-12-02

**Authors:** Tianyu Dai, Junhui Liang, Wei Liu, Yonghui Zou, Feifei Niu, Mengqing Li, Haomeng Zhang, Changzhong Li, Mingjun Fan, Guoying Cui

**Affiliations:** a Department of Gynecology, Shandong Provincial Hospital Affiliated to Shandong First Medical University, Jinan, China; b Department of Gynecology, Shandong Provincial Hospital, Cheeloo College of Medicine, Shandong University, Jinan, China

**Keywords:** miR-582-3p, ovarian cancer, lncRNA TUG1, AKT/mTOR signaling pathway

## Abstract

Ovarian cancer (OC) is one of the most common malignancies of the female reproductive system. The miRNA miR-582-3p is associated with a variety of tumors, and the aim of this study was to investigate the role and mechanisms of miR-582-3p specifically in ovarian carcinogenesis and progression. Low expression of miR-582-3p was noted in OC tissue and cell lines, and lower expression of miR-582-3p correlated with lower overall survival in OC patients. Knockdown of miR-582-3p promoted the proliferation and migration of OC cells, while overexpression inhibited them. TUG1, a long non-coding RNA, was found to bind to miR-582-3p, and inhibition of lncRNA TUG1 decreased viability and migration and weakened the effect of miR-582-3p knockdown in OC cells. Implantation of OC cells with reduced miR-582-3p caused increased tumor growth, while lncRNA TUG1 knockdown suppressed tumor growth and relieved the impact of reduced miR-582-3p *in vivo*. Phosphorylation of AKT and mTOR were significantly enhanced with decreased miR-582-3p expression, but lncRNA TUG1 knockdown attenuated this trend *in vitro* and *in vivo*. The novel miR-582-3p represses the malignant properties of OC *via* the AKT/mTOR signaling pathway by targeting lncRNA TUG1. This axis may represent valuable prognostic biomarkers and therapeutic targets for OC.

## 1. Introduction

Ovarian cancer (OC) is one of the major forms of cancer that affects women’s health worldwide. According to global cancer statistics reports, ovarian cancer is the fifth leading cause of death among women’s tumors, after lung cancer, breast cancer, colorectal cancer and pancreatic cancer [1]. The incidence of OC is undergoing a rapid increase, and the Globocan study predicted that the incidence of OC will increase to 371,000 and the number of deaths to 254,000 worldwide by 2035 [2]. Notably, China and India are predicted to remain the countries with the highest number of ovarian cancer cases [2].

As the ovaries are located deep in the pelvic cavity, early-stage OC patients usually do not have obvious clinical symptoms or only have mild symptoms; therefore, when patients seek medical attention due to symptoms, such as abdominal distension, many of them have already progressed to later stages. In these patients, the tumor has likely spread widely and metastasized in the abdominopelvic cavity, and the cancer cells have embedded in the peritoneum, thus forming multiple nodules [3].

The current treatment for advanced OC remains chemotherapy and tumor cell reduction, and although a large proportion of patients go into remission after first-line treatment, around 70% of OC patients experience tumor recurrence [4]. With advances in diagnosis and treatment, the 5-year survival rate for ovarian cancer has improved over the past three years, but the overall cure rate is still below 40% [5,6]. Therefore, in order to improve the treatment of ovarian cancer patients, we need to better understand the molecular mechanism involved in ovarian carcinogenesis and to explore highly sensitive and specific markers so that we can accurately determine prognosis and individualize treatment for patients.

Recently, the role of non-coding RNAs, including small RNAs and long non-coding RNAs (lncRNAs), in OC has received increasing attention from researchers [7]. MicroRNAs (miRNA) is a kind of noncoding RNA that are typically 18 to 25 Nucleotide length and that have regulatory functions. Various miRNAs regulate cell growth, differentiation, apoptosis and endocrine metabolism, thereby forming a complex and orderly regulatory network that participates in homeostatic balance throughout the body [8–10].

Many miRNAs have been found to act as post-transcriptional regulators of target RNAs in the context of ovarian cancer. For instance, He *et al*. [11] demonstrated an exosome-dependent mechanism by which miR-205 derived from cancer cells regulates tumor angiogenesis and which implicated exosomal miR-205 as a potential therapeutic target for OC. Similarly, An et al [12] found that mir21 It plays an important role in regulating macrophage polarization, there by Increased M2 macrophage mediated chemoresistance of Ovarian cancer cells.

Another miRNA, miR-582-3p, is an important component of the miRNA regulatory network that plays an important management player many diseases [13–17]. Recent data display reported that miR-582-3p plays an important regulatory role in the development and progression of malignant tumors, such as prostate cancer [14], lung cancer [15], liver cancer [16] and cervical cancer [17]. The expression of miR-582-3p in these tumor tissues is often down-regulated, suggesting that miR-582-3p may play a role in suppressing tumourigenesis. The expression of miR-582-3p affects tumor cell reproduction, differentiation and apoptosis through the regulation of multiple target genes, and is a relatively important regulator of malignant tumor cell invasion, vascular infiltration and metastasis. Thus, miR-582-3p has been shown to impact multiple types of cancer, yet few reports exist regarding the role and expression of miR-582-3p specifically in ovarian carcinogenesis.

Recent studies have shown that lncRNAs, which are stable non-coding RNAs greater than 200, can act as upstream regulators by binding and regulating specific miRNAs involved in various biological processes, including tumor cell proliferation, apoptosis, invasion and metastasis [18]. Many lncRNAs have considerable tissue or cell type specificity [19], and lncRNAs as a new participant play in the initiation, maintenance and progression of tumourigenesis [7,20]. For example, like lncrna differentiation against non proteins coding RNA (DANCR) plays a promotional role in tumor angiogenesis in ovarian cancer through regulation of miR-145 [21].

Previous bioinformatic prediction and validation led us to identify lncRNA taurine up-regulated 1 (TUG1), which is upstream of miR-582-3p as another molecule of potential interest in ovarian cancer. It has been found that lncRNA TUG1 is aberrantly expressed in a variety of malignant tumor tissues [22,23]. The aberrantly expressed lncRNA TUG1 interferes with the proliferation, migration and invasion of tumor cells through various molecular mechanisms to promote or inhibit tumor development. The lncRNA TUG1 and specific mRNA can associate through a common miRNA response element, thus forming a competitive endogenous RNA (ceRNA) network. In this network, lncRNA TUG1 attenuates the inhibitory effect of miRNAs on mRNAs by competing for miRNA binding sites [22–24]. This explains to some extent the correlation between genome size and organismal complexity and could provide answers to evolutionary questions, as well as having implications for disease, interpreting disease processes and providing opportunities for new therapies. For instance, TUG1 may contribute to the progression of thyroid cancer cells by functioning as a ceRNA that sponges miR-145 [22].

The pathway that consists of phosphatidylinositol 3-kinase (PI3K), AKT, and mammalian target of rapamycin (mTOR) represents a potential target of non-coding RNA signaling networks, as it regulates cell apoptosis growth, proliferation, differentiation, apoptosis and metabolism, and is the most common altered signaling through the human body tumors [25]. It exerts control over the cell cycle as well as over several important stages of the tumor process, such as angiogenesis, tumor resistance, and genomic instability, and is therefore an important target for anti-tumor therapy.

Among the numerous aberrant oncogenic signaling pathways in ovarian cancer, the PI3K/AKT/mTOR pathway is one of the most frequently altered. In high-grade plasma ovarian cancers, PIK3CA gene amplification mutations were found in more than 20% of cases, and more than 35% of high-grade plasma ovarian cancers presented with combinatorial mutations in components of PI3K/Akt/mTOR signaling pathway, resulting in enhanced pathway activity [26]. Campbell et al. found that amplifying mutations in the PIK3CA gene were reported in 51 (30.5%) of 167 primary epithelial ovarian cancers [27]. Therefore, we investigated the AKT/mTOR signaling pathway as a potential pathway downstream of miR-582-3p activity.

In this study, we focused on miR-582-3p and combined it with its upstream regulator lncRNA TUG1 and potential downstream target AKT/mTOR to explore the malignant biological behavior of this miRNA in regulating ovarian cancer and to analyze the possible molecular mechanisms. Such an analysis may provide new strategies for the treatment of ovarian cancer and new biomarkers for the prediction of ovarian cancer prognoses.

## 2. Materials and methods

### 2.1. Cell culture and plasmid transfection

A2780 and SKOV3 cells were cultured in RPMI-1640 medium supplemented with 10% fetal bovine serum, 100 U/ml penicillin and 100 mg/ml streptomycin. The cells were cultured at 37°C in 5% CO_2_ atmosphere. An miR-582-3p mimic, mimic negative control, miR-582-3p inhibitor, inhibitor negative control and interfering RNA against lncRNA TUG1 were all synthesized by Guangzhou Ribo Biotechnology Co., Ltd.

A2780 and SKOV3 cells were grown in 6-well plates to 70% confluence, when they were transfected with the above plasmids or oligonucleotides using Lipofectamine 2000. The cells were divided into the following groups: control group, untransfected cells; NC-Ov group, cells transfected with the mimic negative control; Ov-miR-582-3p group, cells transfected with an miR-582-3p mimic; NC-si group, cells transfected with an inhibitor negative control; si-miR-582-3p group, cells transfected with miR-582-3p inhibitor; sh-TUG1 group, cells transfected with interfering RNA against lncRNA TUG1; and sh-TUG1+ si-miR-582-3p group. Transfection efficiency was detected by qRT-PCR 48 h after transfection.

### 2.2. Quantitative real-time PCR (qRT-PCR) to determine lncRNA TUG1 and miR-582-3p expression in OC cells

Total RNA was extracted from OC cells using TRIzol reagent. RNA reverse transcription was performed using the FastQuant cDNA First Strand Synthesis Kit, and the obtained complementary DNA (cDNA) was used as the template for PCR. The qRT-PCR assay was performed on an Applied Biosystems 7500 Real-Time PCR System using SuperReal Fluorescent Quantitative Premix Reagent. The relative expressions of lncRNA TUG1 and miR-582-3p were analyzed according to the 2^−ΔΔCt^ method. The primer sequences were as follows: miR-582-3p (F: 5ʹ-GCG CGT AAC TGG TTG AAC AAC-3ʹ; R: 5ʹ-GTC GTA TCC AGT GCA GGG TCC GAG GTA TTC GCA CTG GAT ACG ACG GTT CA-3ʹ), TUG1 (F: 5ʹ-TTC AGC AGG AAG GAT TCA GTT-3ʹ; R: 5ʹ-GCG AGC AGT CTA CAG CAA CC-3ʹ).

### 2.3. CCK-8 assay for detecting cell proliferation

Indicated groups of OC cells at logarithmic growth stage were digested with trypsin and prepared into cell suspensions of suitable concentration. Then, the cell suspension was inoculated at 5 × 10^3^ cells/well in 96-well plates with 100 μL of medium. The plates were foster 24, 48, 72 and 96 hours, and 10 μL CCK-8 added solution to each other well, followed by an additional 1 h incubation. The absorbance at 450 nm was measured with a microplate reader.

### 2.4. Cell clone formation assay

When the indicated group of cells reached logarithmic growth phase, cells were digested with trypsin. Suspended cells were added to 6 cm cell culture dishes at densities of 1500 cells/well, and the plates were put into a 37°C, 5% CO_2_ incubator for 14 days. Then, the cells washed away 2 times with PBS and fixed in 4% paraformaldehyde for a half of one hour. One milliliter of a 0.1% crystal violet staining solution was added to each well, and the plates were stained for 10 min, photographed and counted.

### 2.5. Dual luciferase reporter assay

The lncRNA TUG1 wild-type (WT) or mutant (MUT) sequences containing a putative miR-582-3p binding site were inserted into the pmirGLO dual luciferase vector and named WT-TUG1 or MUT-TUG1, respectively. Then, using Lipofectamine 2000, A2780 co infectious cell with WT-TUG1 or MUT-TUG1 and miR-582-3p mimics or negative controls. Cells were harvested 48 h post-transfection. Luciferase activity was detected using a dual luciferase reporter gene assay system according to the manufacturer’s instructions.

### 2.6. Western blot assays to determine p-AKT, AKT, p-mTOR and mTOR protein expression

Total protein was obtained by lysis in RIPA buffer for indicated group of cells or isolated tumor tissues. Protein lysates were quantified using a BCA Protein Assay Kit, and then separated by SDS-PAGE. The proteins were transferred to PVDF membranes and then incubated with 5% skim milk. The membranes were incubated overnight at 4°C with primary antibodies and for 1 h with secondary antibodies. Primary antibodies included anti-p-AKT (used at a dilution of 1:2000), anti-AKT (1:1000), anti-p-mTOR (1:2000), anti-mTOR (1:5000) and anti-β-actin (1:1000). Secondary antibodies were labeled with horseradish peroxidase and used at a dilution of 1:5000. Signals were observed with an enhanced chemiluminescence system. Protein expression levels were normalized using β-actin as a control.

### 2.7. Analysis of tumorigenesis in nude mice

Twenty 5-week-old SPF-grade female nude mice, weighing approximately 23 g, were randomly divided into four groups and kept in an SPF-grade sterile room with free access to food and water. After transfection, A2780 cells of each group in logarithmic growth phase were made into 1 × 10^7^/mL cell suspensions, and 200 μL of each cell suspension was inoculated under the skin of the left axilla of each group of nude mice, and tumor formation was detected the next day. The weights of the nude mice were recorded, and the length and width of tumors were measured every 7 days, and the tumor volume was calculated according to the equation volume = (length×width^2^)/2. The tumor growth curve was plotted as the transplantation tumor volume in each group. On the 28th day, the mice were sacrificed by cervical dislocation, and the tumors were removed. The experimental procedures involving animals were performed with the approval of the Ethics Committee of Shandong Provincial Hospital Affiliated to Shandong First Medical University (N0.2020–020).

### 2.8. Statistical analysis

Data were analyzed with SPSS 19. 0 statistical software. The measurement data were expressed as mean ± standard deviation, and the paired *t*-test was used for intra-group comparisons that conformed to normal distribution and chi-squaredness, the unpaired *t*-test was used for inter-group comparisons, the one-way ANOVA was used for multi-group comparisons, the LSD-test was used for two-group comparisons, and the rank sum test was used for data that did not conform to normal distribution and chi-squaredness. Differences for which *P < 0.05* were considered statistically significant.

## 3. Results

In this study, low expression of miR-582-3p was noted in OC. Knockdown of miR-582-3p promoted the proliferation and migration of OC cells, while overexpression inhibited them. lncRNA TUG1 was found to bind to miR-582-3p, and inhibition of lncRNA TUG1 decreased viability and migration and weakened the effect of miR-582-3p knockdown in OC cells. Implantation of OC cells with reduced miR-582-3p caused increased tumor growth, while lncRNA TUG1 knockdown suppressed tumor growth and relieved the impact of reduced miR-582-3p *in vivo*. Phosphorylation of AKT and mTOR were significantly enhanced with decreased miR-582-3p expression, but lncRNA TUG1 knockdown attenuated this trend *in vitro* and *in vivo*. The novel miR-582-3p represses the malignant properties of OC *via* the AKT/mTOR signaling pathway by targeting lncRNA TUG1.

### 3.1. Decreased expression of miR-582-3p predicts a the prognosis of patients with ovarian cancer is not ideal

**Figure 1.**
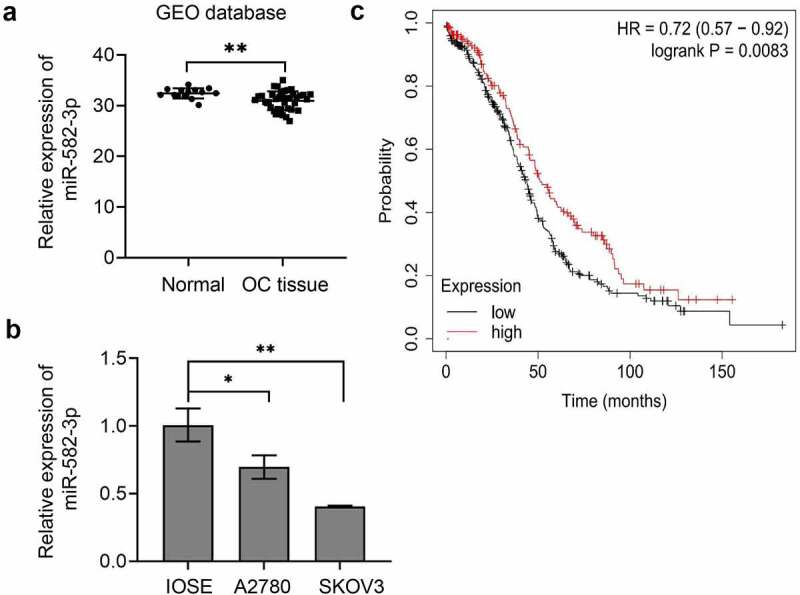
The miRNA miR-582-3p is expressed at relatively low levels in OC and predicts the prognosis in OC patients. (a) The expression of miR-582-3p was evaluated by qRT-PCR analysis of specimens from 39 cases of OC and 14 benign ovarian tissues from the GEO database (GSE53829). (b) Quantitative RT-PCR was performed to detect miR-582-3p expression in OC cell lines (A2780 and SKOV3) and the human ovarian epithelial cell line IOSE. (c) The overall survival of OC patients with high (n = 163) and low (n = 322) miR-582-3p expression was analyzed by the Kaplan-Meier plotter web application, which is based on the Cancer Genome Atlas database. *P < 0.05. Data are presented as mean ± standard deviation (SD)

To determine the importance of miR-582-3p in the development of OC, we first examined the differential expression of miR-582-3p in OC tissues and cell lines. Results of qRT-PCR analyses indicated that the average expression of miR-582-3p in OC tissues was lower than that in benign ovarian tissues (Figure 1(a)), and that miR-582-3p was expressed at a reduced level in OC cell lines compared to the human ovarian epithelial cell line IOSE (Figure 1(b)).

Next, we analyzed the overall survival curve relative to miR-582-3p expression in OC patients. To perform this analysis, we employed the Kaplan-Meier plotter, a TCGA-based tool that is used to assess the effect of genes (mRNA, miRNA, or protein) on survival in a variety of cancers. This analysis demonstrated that the overall survival period was shorter in ovarian cancer patients with low miR-582-3p expression than in those with high miR-582-3p expression (Figure 1(c)).

### 3.2 Decreased miR-582-3p promotes OC cell proliferation and migration in vitro

**Figure 2.**
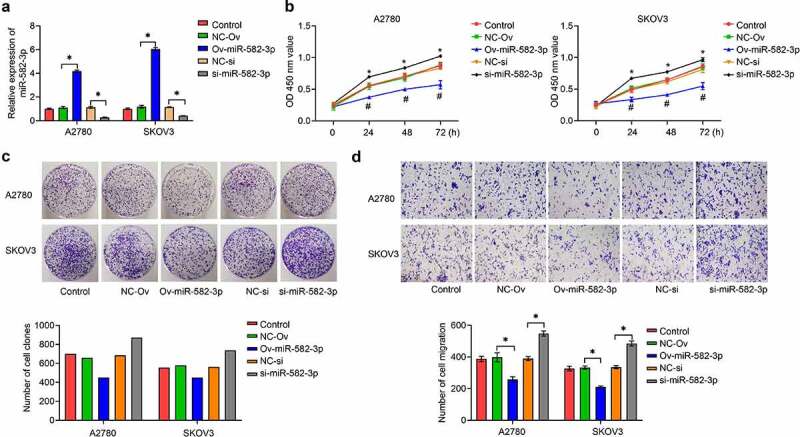
The miRNA miR-582-3p modulates OC cell proliferation and migration *in vitro*. (a) Overexpression and inhibition of expression of miR-582-3p was verified by qRT-PCR assay. (b) CCK-8 and (c) cell clone formation assays were performed to measure cell proliferation. (d) A transwell assay was used to assess cell migratory abilities. *P < 0.05. Data are presented as mean ± SD

To elucidate the function of miR-582-3p in OC, we first achieved overexpression or knockdown of miR-582-3p in OC cells, A2780 and SKOV3, by the use of a miRNA mimic or inhibitor. The overexpression or knockdown effects were validated by qRT-PCR assay (Figure 2(a)). Next, we investigated the effect of miR-582-3p on OC cell proliferation by CCK-8 (Figure 2(b)) and cell clone formation (Figure 2(c)) assays, which showed that overexpression of miR-582-3p inhibited cell proliferation. Conversely, silencing of the expression of miR-582-3p consistently exhibited a promoting impact transwell analysis was used to analyze this problem assessment cell migratory ability and revealed that overexpression of miR-582-3p inhibited migratory capacity, and inhibition of miR-582-3p enhanced it, in A2780 and SKOV3 cells (Figure 2(d)).

### 3.3 The lncRNA TUG1 acts as a molecular sponge for miR-582-3p and regulates miR-582-3p expression

**Figure 3.**
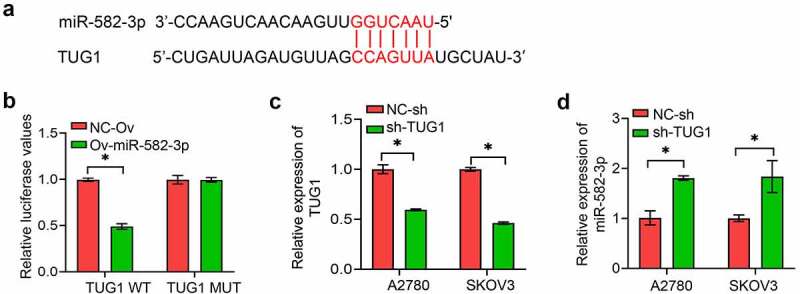
The long non-coding RNA TUG1 acts as a molecular sponge for miR-582-3p. (a) StarBase was used to predict the binding site between lncRNA TUG1 and miR-582-3p. (b) A dual luciferase reporter assay was used to determine the direct correlation between lncRNA TUG1 and miR-582-3p. (c) Inhibition of expression of lncRNA TUG1 was verified by qRT-PCR assay. (d) A qRT-PCR assay was used to evaluate induction of expression of miR-582-3p by knockdown of lncRNA TUG1. *P < 0.05. Data are presented as mean ± SD

To further investigate the underlying mechanism of miR-582-3p, bioinformatics tools were used to analyze correlations between the sequences of various lncRNA and miRNA molecules. We found hypothetical mir-582-3p binding site within lncRNA (Figure 3(a)). This prediction was supported by the results of a dual luciferase reporter assay (Figure 3(b)). Here, overexpression of an miR-582-3p mimic resulted in decreased expression of a luciferase reporter that contained the wild type sequence of lncRNA TUG1 but had no effect on the expression of a reporter with a mutation in the putative binding sequence. Conversely, we used shRNA technology to suppress lncRNA TUG1 expression in A2780 and SKOV3 cells and verified this suppression with qPCR (Figure 3(c)). This attenuation of lncRNA TUG1 expression resulted in the upregulation of miR-582-3p expression (Figure 3(d)).

### 3.4 The oncogenic effect of diminished miR-582-3p expression is attenuated by knockdown of lncRNA TUG1 and activation of the AKT/mTOR signaling pathway

**Figure 4.**
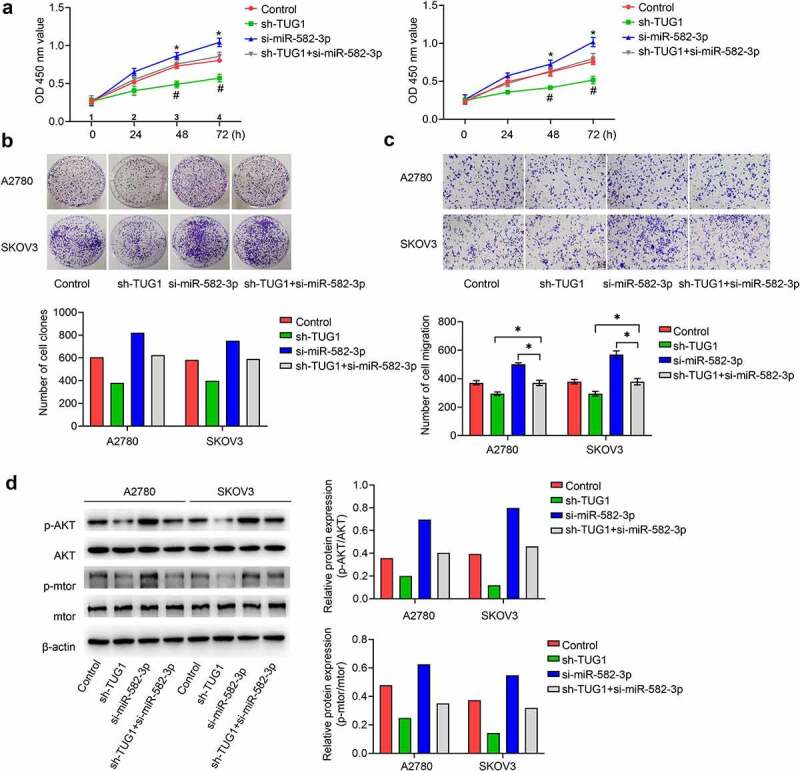
Silenced miR-582-3p promotes the activity of the AKT/mTOR signaling pathway, and this effect is attenuated by lncRNA TUG1 knockdown. (a, b) Cell viability was evaluated with the CCK-8 (a) and cell clone formation (b) assays. (c) The Transwell assay was performed to measure the migration ability. (d) Western blot analysis of p-AKT, AKT, p-mTOR and mTOR from OC cell lysates. *P < 0.05. Data are presented as mean ± SD

We next sought to investigate whether lncRNA TUG was responsible for the enhanced proliferation and migration induced by diminished miR-582-3p. We silenced lncRNA TUG1 in A2780 and SKOV3 cells and found that inhibition of lncRNA TUG1 could abolish the role of decreased miR-582-3p in promoting cell proliferation (Figure 4(a,b)) and migration (Figure 4(c)). Therefore, we conclude that silenced lncRNA TUG1 plays a role in inhibiting cell proliferation and migration ability in OC cells.

As the AKT/mTOR signaling pathway has recently been found to play a critical role in mediating the development of OC, it represents a potential target of these RNA-dependent effects. To address whether the AKT/mTOR signaling pathway is regulated by miR-582-3p, we detected the protein levels of AKT and mTOR and the phosphorylation status of these proteins. Accordingly, signals representing phosphorylated AKT (p-AKT) and p-mTOR were significantly enhanced in cells in which miR-582-3p was under expressed. Further confirming the relationships demonstrated above, knockdown of lncRNA TUG1 attenuated the effect of decreased miR-582-3p expression in OC cells (Figure 4(d)).

### 3.5. *The lncRNA TUG1 decreases miR-582-3p expression and promote test tumor growth in vivo*miR-582-3p on OC growth *in vivo*

**Figure 5.**
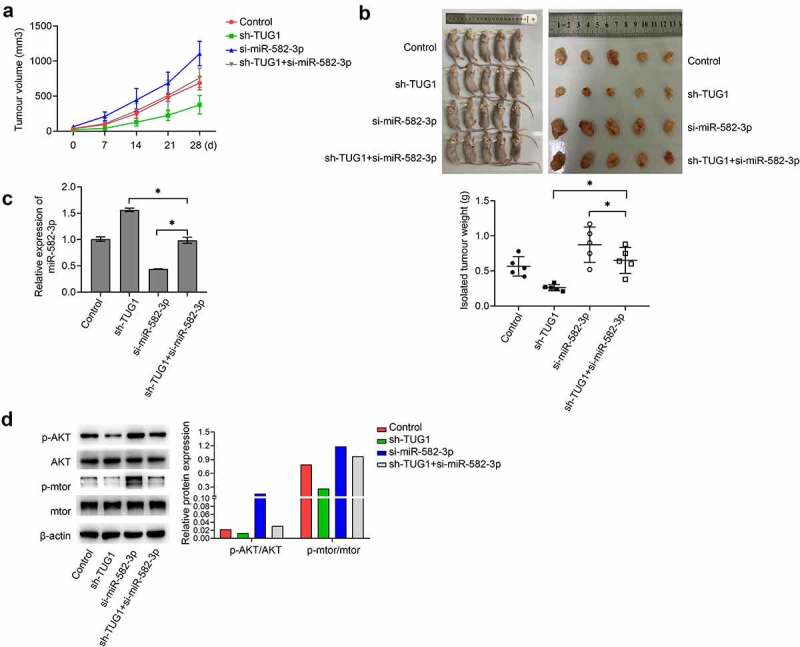
Silenced miR-582-3p promotes tumor growth *in vivo*. (a) Tumor volume was measured at various time points as indicated. (b) Isolated tumor weight was determined after animals were sacrificed (n = 5). (c) The expression of miR-582-3p in tumors was evaluated by qRT-PCR analyses. (d) Western blot analysis of p-AKT, AKT, p-mTOR and mTOR from isolated tumors. *P < 0.05. Data are presented as mean ± SD

Nude mice were implanted with A2780 cells that were expressing different levels of miR-582-3p and lncRNA TUG1. Analyses of the subsequent tumors demonstrated that silenced miR-582-3p caused a significantly increased tumor growth, including tumor volume and weight; however, knockdown of lncRNA TUG1 remarkably suppressed these effects. Moreover, as compared with tumors under expressing only miR-582-3p, tumors in which both miR-582-3p and lncRNA TUG1 were knocked down showed increased tumor volume and weight (Figure 5(a,b)). In addition, implantation of OC cells with silenced miR-582-3p generated tumors with lower expression of miR-582-3p, while lncRNA TUG1 knockdown caused enhanced expression of miR-582-3p and relieved the impact of reduced miR-582-3p (Figure 5(c)). Phosphorylation of AKT and mTOR *in vivo* exhibited a trend similar to that observed *in vitro* in that lower expression of miR-582-3p in tumors correlated with enhanced activity in this pathway, and this effect was offset by knockdown of lncRNA TUG1 (Figure 5(d)).

## 4. Discussion

Although some molecular mechanisms of the initiation and progression of OC have been elucidated in recent years, especially regarding novel roles of miRNA [11,12,28], mechanisms of progression of this important disease are still incompletely understood. The roles of miRNA are of particular interest, as dysregulation of miRNA metabolism is involved in almost every process leading to carcinogenesis and progression, including proliferation, apoptosis, migration, invasion, angiogenesis, and metastasis [8–10]. Accordingly, many studies have evaluated the expression profiles of miRNAs in tissue and serum samples from OC patients to find appropriate biomarkers for this malignancy. Functional assays have also confirmed the oncogenic or inhibitory effects of several miRNAs in OC. As miRNAs have such a wide range of potential functions, we expect that additional biomarkers and therapeutic targets will be identified in the near future. In the present research, we concentrated on miR-582-3p and aligned it with its upstream regulator lncRNA TUG1 and downstream target, the AKT/mTOR signaling pathway, to explore the behavior of this miRNA in the biology of ovarian cancer.

Studies of miRNAs have demonstrated two different patterns of changes in miRNA expression in cancer, specifically increased and decreased expression, and different miRNAs function as tumor suppressor genes or oncogenes in different settings [29,30]. Therefore, we first examined miR-582-3p expression in OC cell lines, and the results showed that miR-582-3p was expressed at relatively low levels in two OC cell lines, A2780 and SKOV3. This finding is consistent with the trend of low miR-582-3p expression in prostate cancer and liver cancer that has been reported [14,16]. Next, based on a large sample of cases in the TCGA database, we determined that low expression of miR-582-3p in OC correlated with lower survival times. These results suggest that miR-582-3p functions as a tumor suppressor gene in OC and that strategies to alter this dysregulated expression may have positive clinical implications.

To investigate the possible mechanism by which low expression of miR-582-3p acts as a tumor suppressor gene in OC, we achieved overexpression or silencing of expression of miR-582-3p in OC cells through the transfection of miRNA mimics or inhibitors, and then examined the effects of these alterations on the proliferation and migration of ovarian cancer cells. Cell proliferation is closely related to tumor development, and one of the characteristics of malignant tumor cells is the ability to proliferate indefinitely. The generation and release of pro-cell growth signals in normal tissues can be precisely controlled, thus ensuring that cell proliferation and differentiation proceed cyclically and maintaining a balance in cell numbers. However, the continuous production of cell proliferation signals are one of the main features of malignant tumors, which cannot achieve negative feedback regulation. This aberrant production is manifest as unlimited division and proliferation and continuous stimulation of proliferative signals [31]. Here, the results of CCK-8 and cell clone formation experiments showed that overexpression of miR-582-3p inhibited OC cell proliferation, and accordingly, silencing of expression of miR-582-3p exhibited a promoting impact.

Cell migration is another key component of pathological processes, such as local dissemination of malignant tumors, and abnormal expression of adhesion molecules within tumor cells or defective development of the cytoskeleton can cause changes in their migratory behavior. It is particularly important to study cell migration in OC because the pathway of OC metastasis is mainly abdominopelvic dissemination and implantation metastasis, and most patients have already developed extensive intra-abdominal metastases at the time of consultation [32]. The transwell analysis was used to analyze and evaluate the cell migratory ability and revealed that overexpression of miR-582-3p inhibited migratory capacity, and inhibition of miR-582-3p enhanced it in A2780 and SKOV3 cells. Taken together, these results suggest that miR-582-3p inhibits the proliferation and migratory capacity of ovarian cancer cells *in vitro*.

Two types of non-coding RNAs, lncRNAs and miRNAs, form complex regulatory networks, and we next identified and validated the miR-582-3p-binding ability of an lncRNA. By bioinformatic prediction, we identified lncRNA TUG1 as a potential partner and validated the binding between lncRNA TUG1 and miR-582-3p based on dual luciferase assays. Numerous studies have reported that lncRNAs can act as ceRNAs, recruiting bound miRNAs through the ‘sponge adsorption effect’ and inhibiting the functions of the target miRNAs [22–24]. To test the applicability of this mechanism to the miR-582-3p/TUG1 relationship, we designed a ‘functional backfill’ experiment. The results showed that inhibition of lncRNA TUG1 could abolish the role of decreased miR-582-3p in promoting cell proliferation and migration ability. Meanwhile, silencing of lncRNA TUG1 alone plays a role in inhibiting cell proliferation and migration ability in OC cells. These results are consistent with a model in which lncRNA TUG1 promotes ovarian carcinogenesis through competitive inhibition of miR-582-3p.

Given that the PI3K/AKT/mTOR signaling pathway is frequently mutated in ovarian cancer, we further examined whether the pathway was regulated by miR-582-3p. The results showed that the phosphorylation of AKT and mTOR were significantly enhanced in cells with miR-582-3p under-expression. However, lncRNA TUG1 knockdown attenuated this trend, confirming that the AKT/mTOR signaling pathway was further activated when low expression of miR-582-3p exerted a pro-oncogenic effect. In addition, we verified the function and role of miR-582-3p *in vivo* by tumorigenic assays in nude mice. The results were consistent with *in vitro* results, indicating that miR-582-3p can bind to the upstream regulator lncRNA TUG1 and affect the activation state of the downstream AKT/mTOR signaling pathway.

In summary, in this study, we demonstrated that the expression level of mir-582-3p was relatively lowin ovarian cancer cells and that low miR-582-3p expression in patients with ovarian cancer with worse survival. Knockdown of miR-582-3p promotes the proliferation and migration ability of ovarian cancer and activates the downstream AKT/mTOR signaling pathway, and this promotion is partially abrogated by the knockdown of upstream lncRNA TUG1.

## 5. Conclusion

The correlations of miR-582-3p expression and its interactions with lncRNA TUG1 with AKT/mTOR signaling and ovarian cancer outcomes suggest that the lncRNA TUG1/miR-582-3p/AKT/mTOR axis may be used as a novel prognostic biomarker and therapeutic target in OC.

## Highlights

Low expression of miR-582-3p was noted in OC.

miR-582-3p represses the malignant properties of OC via the AKT/mTOR signaling pathway.

miR-582-3p targets lncRNA TUG1.

## References

[1] Siegel RL, Miller KD, Jemal A. Cancer statistics. CA Cancer J Clin. 2019;69(1):7–34. 30620402 10.3322/caac.21551

[2] Bray F, Ferlay J, Soerjomataram I, et al. Global cancer statistics 2018: GLOBOCAN estimates of incidence and mortality worldwide for 36 cancers in 185 countries. CA Cancer J Clin. 2018;68(6):394–424. 30207593 10.3322/caac.21492

[3] Webb PM, Jordan SJ. Epidemiology of epithelial ovarian cancer. Best Pract Res Clin Obstet Gynaecol. 2017;41:3–14. 27743768 10.1016/j.bpobgyn.2016.08.006

[4] Orr B, Edwards RP. Diagnosis and treatment of ovarian cancer. Hematol Oncol Clin North Am. 2018;32(6):943–964. 30390767 10.1016/j.hoc.2018.07.010

[5] Eisenhauer EA. Real-world evidence in the treatment of ovarian cancer. Ann Oncol. 2017;28(suppl_8):viii61–viii65. 29232466 10.1093/annonc/mdx443

[6] Narod S. Can advanced-stage ovarian cancer be cured? Nat Rev Clin Oncol. 2016;13(4):255–261. 26787282 10.1038/nrclinonc.2015.224

[7] Zhan L, Li J, Wei B. Long non-coding RNAs in ovarian cancer. J Exp Clin Cancer Res. 2018;37(1):120. 29921308 10.1186/s13046-018-0793-4 PMC6008930

[8] Andersen GB, Tost J. Circulating miRNAs as biomarker in cancer. Recent Results Cancer Res. 2020;215:277–298. 31605235 10.1007/978-3-030-26439-0_15

[9] Tutar L, Özgür A, Tutar Y. Involvement of miRNAs and pseudogenes in cancer. Methods Mol Biol. 2018;1699:45–66. 29086367 10.1007/978-1-4939-7435-1_3

[10] Saliminejad K, Khorram Khorshid HR, Soleymani Fard S, et al. An overview of microRNAs: biology, functions, therapeutics, and analysis methods. J Cell Physiol. 2019;234(5):5451–5465. 30471116 10.1002/jcp.27486

[11] He L, Zhu W, Chen Q, et al. Ovarian cancer cell-secreted exosomal miR-205 promotes metastasis by inducing angiogenesis. Theranostics. 2019;9(26):8206–8220. 31754391 10.7150/thno.37455 PMC6857047

[12] An Y, Yang Q. MiR-21 modulates the polarization of macrophages and increases the effects of M2 macrophages on promoting the chemoresistance of ovarian cancer. Life Sci. 2020;242:117162. 31837336 10.1016/j.lfs.2019.117162

[13] He J, Su X, Xie W. MiR-582-3p alleviates osteoarthritis progression by targeting YAP1. Mol Immunol. 2020;128:258–267. 33190006 10.1016/j.molimm.2020.10.022

[14] Huang S, Zou C, Tang Y, et al. miR-582-3p and miR-582-5p suppress prostate cancer metastasis to bone by repressing TGF-β signaling. Mol Ther Nucleic Acids. 2019;16:91–104. 30852380 10.1016/j.omtn.2019.01.004 PMC6409413

[15] Fang L, Cai J, Chen B, et al. Aberrantly expressed miR-582-3p maintains lung cancer stem cell-like traits by activating Wnt/β-catenin signalling. Nat Commun. 2015;6(1):8640. 26468775 10.1038/ncomms9640 PMC4667703

[16] Zhang H, Dai Q, Zheng L, et al. Knockdown of circ_HIPK3 inhibits tumorigenesis of hepatocellular carcinoma via the miR-582-3p/DLX2 axis. Biochem Biophys Res Commun. 2020;533(3):501–509. 32977948 10.1016/j.bbrc.2020.09.050

[17] Xu J, Zhang Y, Huang Y, et al. circEYA1 functions as a sponge of miR-582-3p to suppress cervical adenocarcinoma tumorigenesis via upregulating CXCL14. Mol Ther Nucleic Acids. 2020;22:1176–1190. 33312754 10.1016/j.omtn.2020.10.026 PMC7701031

[18] Paraskevopoulou MD, Hatzigeorgiou AG. Analyzing miRNA-lncRNA interactions. Methods Mol Biol. 2016;1402:271–286. 26721498 10.1007/978-1-4939-3378-5_21

[19] Jarroux J, Morillon A, Pinskaya M. History, Discovery, and Classification of lncRNAs. Adv Exp Med Biol. 2017;1008:1–46. 28815535 10.1007/978-981-10-5203-3_1

[20] Bhardwaj V, Tan YQ, Wu MM, et al. Long non-coding RNAs in recurrent ovarian cancer: theranostic perspectives. Cancer Lett. 2021;502:97–107. 33429007 10.1016/j.canlet.2020.12.042

[21] Lin X, Yang F, Qi X, et al. LncRNA DANCR promotes tumor growth and angiogenesis in ovarian cancer through direct targeting of miR-145. Mol Carcinog. 2019;58(12):2286–2296. 31545000 10.1002/mc.23117

[22] Lei H, Gao Y, Xu X. LncRNA TUG1 influences papillary thyroid cancer cell proliferation, migration and EMT formation through targeting miR-145. Acta Biochim Biophys Sin (Shanghai). 2017;49(7):588–597. 28645161 10.1093/abbs/gmx047

[23] Zong M, Feng W, Wan L, et al. TUG1 promotes esophageal cancer development through regulating PLK1 expression by sponging miR-1294. Biotechnol Lett. 2020;42(12):2537–2549. 33009634 10.1007/s10529-020-02984-0

[24] Yan Z, Bi M, Zhang Q, et al. LncRNA TUG1 promotes the progression of colorectal cancer via the miR-138-5p/ZEB2 axis. Biosci Rep. 2020;40(6):BSR20201025. 32391554 10.1042/BSR20201025 PMC7280475

[25] Alzahrani AS. PI3K/Akt/mTOR inhibitors in cancer: at the bench and bedside. Semin Cancer Biol. 2019;59:125–132. 31323288 10.1016/j.semcancer.2019.07.009

[26] Altomare DA, Wang HQ, Skele KL, et al. AKT and mTOR phosphorylation is frequently detected in ovarian cancer and can be targeted to disrupt ovarian tumor cell growth. Oncogene. 2004;23(34):5853–5857. 15208673 10.1038/sj.onc.1207721

[27] Campbell IG, Russell SE, Choong DY, et al. Mutation of the PIK3CA gene in ovarian and breast cancer. Cancer Res. 2004;64(21):7678–7681. 15520168 10.1158/0008-5472.CAN-04-2933

[28] Deb B, Uddin A, Chakraborty S. miRNAs and ovarian cancer: an overview. J Cell Physiol. 2018;233(5):3846–3854. 28703277 10.1002/jcp.26095

[29] Qadir MI, Faheem A. miRNA: a diagnostic and therapeutic tool for pancreatic cancer. Crit Rev Eukaryot Gene Expr. 2017;27(3):197–204. 29199604 10.1615/CritRevEukaryotGeneExpr.2017019494

[30] Vishnoi A, Rani S. miRNA biogenesis and regulation of diseases: an overview. Methods Mol Biol. 2017;1509:1–10. 27826912 10.1007/978-1-4939-6524-3_1

[31] White E, Mehnert JM, Chan CS. Autophagy, metabolism, and cancer. Clin Cancer Res. 2015;21(22):5037–5046. 26567363 10.1158/1078-0432.CCR-15-0490 PMC4646728

[32] Yeung TL, Leung CS, Yip KP, et al. Cellular and molecular processes in ovarian cancer metastasis. A review in the theme: cell and molecular processes in cancer metastasis. Am J Physiol Cell Physiol. 2015;309(7):C444–56. 26224579 10.1152/ajpcell.00188.2015 PMC4593771

